# Challenges of Teaching Physiology in an Integrated System-Based Curriculum

**Published:** 2012-03-31

**Authors:** Zuheir Hasan, Reginald Sequeira

**Affiliations:** 1 Faculty of Medicine, Jordan University of Science & Technology, Irbid, Jordan; 2 WHO Centre for Educational Development, CMMS, Arabian Gulf University, Kingdom of Bahrain

## Abstract

The transformation of a traditional discipline-based medical curriculum into a system-based integrated curriculum often poses dilemmas to faculty involved in teaching basic medical sciences. This paper examines the challenges of teaching physiology to medical students in a system-based curriculum. Some of these challenges include: defining the core curriculum, curriculum links, sequencing curriculum content, interdisciplinary integration, and student assessment. A number of relevant issues including defining the core physiology content, faculty expertise, and coping and adapting to curriculum transitions are discussed from a personal perspective. For successful implementation of a system-based curriculum and to overcome the challenges, educational issues should be debated in regional and international forums.

## Introduction

**Figure 1:**
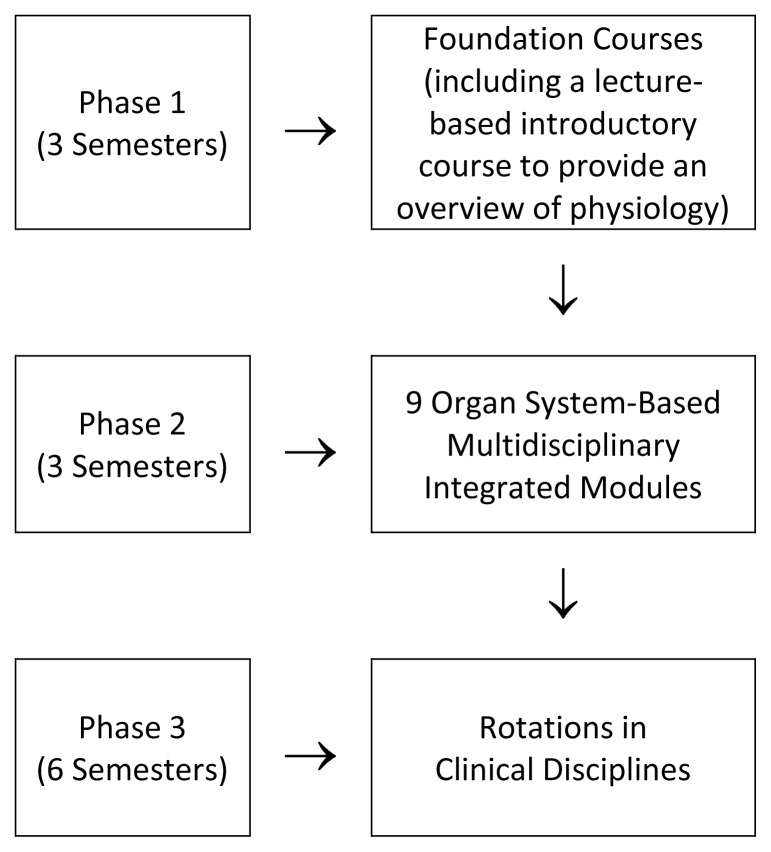
Physiology Inputs in the Revised Curriculum.

Several medical schools in recent decades have introduced integrated system-based curricula.^5^,^6^,^8^ The Faculty of Medicine established in 1983 at Jordan University of Science and Technology undertook a major curriculum revision in 2001. The traditional lecture-based, departmentally segmented pre-clerkship curriculum was redesigned to enhance horizontal and vertical integrations. The physiology curriculum is now organized around nine organ-based modules and is preceded by a foundation course (Figure 1).

The curriculum transformation encountered resistance because the well-structured courses in basic sciences have been eliminated, and implementation faced challenges, especially in basic medical sciences. Problems and challenges of integrated medical curricula in many medical schools have been described by others.^1^,^3^,^4^,^7^,^10^,^11^ We present our personal views, evolved over years of teaching in a system-based curriculum, traditional curriculum (and PBL curriculum), and based on informal feedback from stakeholders.

### How were physiology learning objectives determined? Are there differences in learning objectives in system-based versus traditional curricula?

In a traditional curriculum the course specifications are determined by faculty of the physiology department. However, in a system-based integrated curriculum the decisions about curriculum content are determined by faculty from clinical and basic disciplines, and are based on community health problems. The health problems are selected on the basis of criteria such as prevalence, interdisciplinary nature, preventability, emergencies, and conditions that illustrate basic science concepts. The specific learning objectives of physiology and other basic medical sciences emerge through the problems. For example, the learning objectives related to the electrophysiology of the heart include understanding palpitations, correlating anatomy of the conduction system and pathophysiology of arrhythmias, the types and causes of arrhythmias, and management principles are embedded to enhance integration. Such an approach emphasizes the basic science concepts in a clinical context, and hence evoke student’s interest, makes the learning experience enjoyable, and encourages knowledge application.^2^,^7^,^9^

Qualitatively there is perhaps little difference in the core knowledge or skills students acquire in both curricula. The difference is in how the learning objectives are identified and achieved. In a system-based curriculum, the learning is needs based, conceptual, and problem related. Lectures emphasize relevant concepts and sensitize the students to supplementary learning resources, thereby encouraging self-directed learning. The learning is further enhanced by concurrent lectures delivered by clinical faculty to address the clinical dimensions of the health problems. Participation by clinical faculty in developing and implementing the curriculum enhances the integration of basic and clinical sciences and emphasizes the relevance of basic science content to clinical encounters.

### Does the system-based curriculum allocate enough time for physiology?

In our traditional curriculum, students were given 145 lectures, and laboratory sessions were carried out by students, with the aim of introducing basic clinical skills. In the revised curriculum, physiology lectures have been reduced by more than 50 percent but skills remain essentially the same. The time constraint caused difficulties for both faculty and students; many faculty felt that some complex physiological concepts are not adequately addressed.

With the introduction of an integrated curriculum more time is built into the academic schedule for independent study, faculty are expected to use the scheduled lectures to provide a conceptual framework of physiological principles and provide directions for independent study. Clinical scenarios can be presented in a small-group learning setting to further enhance important physiological concepts, for example, pulmonary embolism to illustrate ventilation/perfusion imbalance.

### Are some of the basic physiology concepts marginalized?

Faculty participating in physiology teaching have the impression that some important concepts are not adequately addressed, a perception often influenced by faculty expertise and expectations. It is likely that some concepts may be marginalized, an observation that may well have gone unnoticed. Curriculum review groups should agree on the core curriculum and outcomes expected.

### Are there unique challenges for teaching physiology due to the sequence of the curriculum?

Whereas a traditional curriculum logically explores the complexity of organ functions, in an integrated curriculum topics cannot always be sequenced to provide students with the prerequisite knowledge for understanding more complex topics. The issue of sequencing topics poses a challenge to physiology teachers.

The lecture-based foundation course in physiology (Figure 1) is intended to provide an overview of basic cellular physiology, autonomic nervous system, nerve and muscle, synaptic transmission, body fluid compartments, and an overview of the physiology of organ systems. Once new related concepts are introduced in the modules, students are expected to review and apply these concepts to the new knowledge. Revisiting concepts at greater depth is encouraged through a spiral curriculum design.

### Are the physiology learning objectives adequately assessed in tests?

The method of student assessment and the evaluation of the learning outcomes include multiple choice questions (MCQs) and OSPE exams to test knowledge and skills, respectively. The test blueprints are constructed based on learning objectives of the modules, determined by a module coordinator and participating faculty. Writing integrated test items is one of the major challenges faced by faculty who are used to writing discipline-specific test items. This problem was partially resolved by writing cluster MCQs based on clinical vignettes, which are peer reviewed.

Due to limited sampling of test items, a comprehensive coverage was deemed difficult. However, discipline representation in exams is not totally lost because the number of test items allocated for each discipline is proportional to curriculum inputs. Nevertheless, faculty and student dissatisfaction about content coverage was not unusual, even in the traditional curriculum: test items often tested factual knowledge unlikely to be recalled or applied in the future or not relevant to the competencies expected from graduating physicians. This limitation in student assessment has been partially addressed by ensuring that mainly the core learning objectives are assessed.

### Does the system-based curriculum achieve true integration of physiology?

Medical students are expected to benefit when the knowledge of diseases is presented with multidisciplinary inputs blended together through integration. Some faculty described many missed opportunities for achieving true integration; some concepts are repeated, while others are never introduced. This discrepancy stems from the fact that some faculty did not have a comprehensive understanding of the medical curriculum beyond their area of specialization. Few made efforts to know the curriculum details or communicated with faculty participating in other modules. Therefore, they had little idea of what students had previously studied and delivered their lectures without these insights. In some instances, lectures used in teaching physiology in a traditional curriculum were delivered in the system-based modules with minimal changes. Often faculty did not succeed in linking their discipline content with the rest of the curriculum, resulting in repetition, redundancies, and gaps in student’s knowledge. These implementation issues are likely due to communication gaps and heavy teaching load. It is also important to invest in faculty training and development.

### Do students learn physiology at sufficient depth?

Although the learning objectives are given to students at the commencement of each module, faculty feel that the students are not learning physiology at adequate depth, and that many students have the dilemma of how much detail they need to know. Such a dilemma indicates that these students have difficulty identifying the depth of their learning and this can result in superficial learning. Perhaps, this issue is not related to the curriculum structure, but rather is due to the curriculum implementation strategy and because of faculty perception that detailed content coverage is essential during lectures. Often, faculty expect the students to have prior background knowledge to enable them to reach the point at which they can fully understand physiological concepts. However, this approach to learning is likely to be counterproductive because students are not given the chance to identify their learning needs themselves, thus compromising the process of learning through discovery.

It is important for faculty to realize that the lectures are intended to provide students with a conceptual framework, as a learning scaffold, rather than to deliver factual information. Faculty can facilitate student learning by providing resources for students to acquire and organize knowledge. It is important to encourage interactive teaching and to promote active learning while avoiding information overload.

In conclusion, there is no single ideal curriculum for teaching physiology (and other basic medical sciences), and there is no general consensus on the most appropriate strategy for teaching physiology to medical students. It is imperative that some form of monitoring be adopted to address some of the concerns of the basic science faculty. To what extent can medical students apply these concepts in a clinical context and be able to solve the problem? It is hoped that faculty actively participate in defining the core curriculum, evaluation of teaching strategies, and curriculum revision. Only then can faculty design an appropriate program and fulfill their educational responsibilities. Physiology faculty need to share their experience with both the regional and international fraternity. This may help us learn from each other, and together, we can enhance the effectiveness of student learning.
